# Certifying Proofs in the First-Order Theory of Rewriting

**DOI:** 10.1007/978-3-030-72013-1_7

**Published:** 2021-02-26

**Authors:** Fabian Mitterwallner, Alexander Lochmann, Aart Middeldorp, Bertram Felgenhauer

**Affiliations:** 8 Eindhoven University of Technology, Eindhoven, The Netherlands; 9 Aalborg University, Aalborg East, Denmark; 10 Department of Computer Science, University of Innsbruck, Innsbruck, Austria; 11 Innsbruck, Austria

## Abstract

The first-order theory of rewriting is a decidable theory for linear variable-separated rewrite systems. The decision procedure is based on tree automata techniques and recently we completed a formalization in the Isabelle proof assistant. In this paper we present a certificate language that enables the output of software tools implementing the decision procedure to be formally verified. To show the feasibility of this approach, we present FORT-h, a reincarnation of the decision tool FORT with certifiable output, and the formally verified certifier FORTify.

## 1 Introduction

Many properties of rewrite systems can be expressed as logical formulas in the first-order theory of rewriting. This theory is decidable for the class of linear variable-separated rewrite systems, which includes all ground rewrite systems. The decision procedure is based on tree automata techniques and goes back to Dauchet and Tison [7]. It is implemented in FORT [17,18]. FORT takes as input one or more rewrite systems $\mathcal{R}_0, \mathcal{R}_1, \ldots$ and a formula $\varphi$, and determines whether or not the rewrite systems satisfy the property expressed by $\varphi$, in which case it reports yes or no. FORT may not reach a conclusion due to limited resources.

For properties related to confluence and termination, designated competitions (CoCo [15], termCOMP [9]) of software tools take place regularly. Occasionally, yes/no conflicts appear. Since the participating tools typically couple a plethora of techniques with sophisticated search strategies, human inspection of the output of tools to determine the correct answer is often not feasible. Hence certified categories were created in which tools must output a formal certificate. This certificate is verified by CeTA [21], an automatically generated Haskell program using the code generation feature of Isabelle. This requires not only that the underlying techniques are formalized in Isabelle, but the formalization must be executable for code generation to apply. During the time-consuming formalization process, mistakes in papers are sometimes brought to light.

* This research is supported by FWF (Austrian Science Fund) project P30301.

Since 2017 we are concerned with the question of how to ensure the correctness of the answers produced by FORT. The certifier CeTA supports a great many techniques for establishing concrete properties like termination and confluence, but the formalizations in the underlying Isabelle Formalization of Rewriting (IsaFoR)[3] are orthogonal to the ones required for supporting the decision procedure underlying FORT. We recently completed the formalization of the automata constructions involved in the decision procedure [14]. Earlier fragments were described in [8, 13]. In this paper we put these efforts to the test. More precisely, we

1. present a certificate language which is rich enough to express the various automata operations in decision procedures for the first-order theory of rewriting as well as numerous predicate symbols that may appear in formulas in this theory,
2. describe the tasks required to turn the formalization described in [14] into verified code to check certificates within reasonable time,
3. present a new reincarnation of FORT in Haskell, named FORT-h, which is capable of producing certificates.

The remainder of the paper is organized as follows. The next section briefly recapitulates the first-order theory of rewriting and the variant of the decision procedure described in [14]. Sections 3 and 4 describe the representation of formulas in certificates and the certificate language. In Section 5 we describe how certificates are validated by FORTify, the verified Haskell program obtained from the Isabelle formalization. Section 6 describes FORT-h. Experimental results are presented in Section 7, before we conclude in Section 8.

## 2 Preliminaries

Familiarity with term rewriting [2] and tree automata [6] is useful, but we briefly recall important definitions and notation that we use in the remainder.

Terms $\mathcal{T}(\mathcal{F},\mathcal{V})$ are constructed from a signature $\mathcal{F}$, consisting of function symbols with fixed arities, and a set of variables $\mathcal{V}$. A term rewrite system (TRS for short) $\mathcal{R}$ consists of rewrite rules $\ell \to r$ between terms $\ell$ and $r$. Instead of the usual restrictions $\ell \notin \mathcal{V}$ and $\mathcal{V}\mathsf{ar}(r) \subseteq \mathcal{V}\mathsf{ar}(\ell)$, we require $\mathcal{V}\mathsf{ar}(\ell) \cap \mathcal{V}\mathsf{ar}(r) = \varnothing$. Here $\mathcal{V}\mathsf{ar}(t)$ denotes the set of variables in a term $t$. Moreover, $\ell$ and $r$ are assumed to be linear terms (i.e., variables occur at most once). The conditions on the rewrite rules are necessary to ensure decidability of the first-order theory of rewriting for these *linear variable-separated* TRSs. The (one-step) rewrite relation of a TRS $\mathcal{R}$ is denoted by $\to_{\mathcal{R}}$. A term $t$ is ground if $\mathcal{V}\mathsf{ar}(t) = \varnothing$. The set of ground terms is denoted by $\mathcal{T}(\mathcal{F})$.

The first-order theory of rewriting is defined over a language $\mathcal{L}$ containing the predicate symbols $\to$, $\to^*$, $=$, and many more. As models, we consider finite linear variable-separated TRSs $\mathcal{R}$ over signatures $\mathcal{F}$ such that $\mathcal{T}(\mathcal{F})$ is non-empty. The set $\mathcal{T}(\mathcal{F})$ serves as domain for the variables in formulas over $\mathcal{L}$. The

[3] http://cl-informatik.uibk.ac.at/software/ceta/

interpretation of the predicate symbol $\to$ in $\mathcal{R}$ is the one-step rewrite relation $\to_{\mathcal{R}}$ over $\mathcal{T}(\mathcal{F})$, $\to^*$ denotes the restriction of $\to_{\mathcal{R}}^*$ to terms in $\mathcal{T}(\mathcal{F})$, and $=$ is interpreted as the identity relation on $\mathcal{T}(\mathcal{F})$. Since we use ground terms as carrier, formulas in the first-order theory of rewriting express properties on ground terms. For instance, the following formula $\varphi$ expresses the property of having unique normal forms (UNR):

$$\forall\, s\, \forall\, t\, \forall\, u\, (s \to^* t \wedge \neg \exists\, v\, (t \to v) \wedge s \to^* u \wedge \neg \exists\, v\, (u \to v) \implies t = u)$$

To use $\varphi$ for establishing UNR for arbitrary terms (i.e., terms in $\mathcal{T}(\mathcal{F},\mathcal{V})$) two additional constant symbols need to be added to the signature [18]. (More on this in Section 8.) Additional predicates in $\mathcal{L}$ increase the expressive power and also allow expressing properties more compactly. For instance, we can write $\mathsf{NF}(t)$ for $\neg\exists\, v\, (t \to v)$ and $s \to^! t$ for $s \to^* t \wedge \neg\exists\, v\, (t \to v)$. In Section 3 we present a grammar that describes the available constructions for predicates. All predicates that can be represented using these constructions are supported in our decision procedure.

The decision procedure is based on tree automata that recognize relations on ground terms. Here we give a brief summary. More information can be found in [6] and [14]. A tree automaton $\mathcal{A} = (\mathcal{F}, Q, Q_f, \Delta)$ consists of a finite signature $\mathcal{F}$, a finite set $Q$ of states, disjoint from $\mathcal{F}$, a subset $Q_f \subseteq Q$ of final states, and a set of transition rules $\Delta$. Transition rules have one of the following two shapes: $f(p_1,\ldots,p_n) \to q$ with $f \in \mathcal{F}$ and $p_1,\ldots,p_n,q \in Q$, or $p \to q$ with $p,q \in Q$. The latter are called epsilon transitions. Transition rules can be viewed as rewrite rules between ground terms in $\mathcal{T}(\mathcal{F} \cup Q)$. The induced rewrite relation is denoted by $\to_\Delta$ or $\to_\mathcal{A}$. A ground term $t \in \mathcal{T}(\mathcal{F})$ is accepted by $\mathcal{A}$ if $t \to_\Delta^* q$ for some $q \in Q_f$. The set of all accepted terms is denoted by $L(\mathcal{A})$ and a set $L$ of ground terms is regular if $L = L(\mathcal{A})$ for some tree automaton $\mathcal{A}$.

We encode $n$-tuples with $n \geqslant 1$ of ground terms as terms over an enriched signature, as follows. We write $\mathcal{F}^{(n)}$ for the signature $(\mathcal{F} \cup \{\bot\})^n$ where $\bot \notin \mathcal{F}$ is a fresh constant. The arity of a symbol $f_1 \cdots f_n \in \mathcal{F}^{(n)}$ is the maximum of the arities of $f_1,\ldots,f_n$. The encoding of terms $t_1,\ldots,t_n \in \mathcal{T}(\mathcal{F})$ is the unique term $\langle t_1,\ldots,t_n\rangle \in \mathcal{T}(\mathcal{F}^{(n)})$ such that $\mathcal{P}\mathsf{os}(\langle t_1,\ldots,t_n\rangle) = \mathcal{P}\mathsf{os}(t_1) \cup \cdots \cup \mathcal{P}\mathsf{os}(t_n)$ and $\langle t_1,\ldots,t_n\rangle(p) = f_1 \cdots f_n$ where $f_i = t_i(p)$ if $p \in \mathcal{P}\mathsf{os}(t_i)$ and $f_i = \bot$ otherwise, for all $p \in \mathcal{P}\mathsf{os}(\langle t_1,\ldots,t_n\rangle)$ and $1 \leqslant i \leqslant n$. As an example, for the terms $s = \mathsf{f}(\mathsf{g}(\mathsf{a}),\mathsf{f}(\mathsf{b},\mathsf{b}))$, $t = \mathsf{g}(\mathsf{g}(\mathsf{a}))$, and $u = \mathsf{f}(\mathsf{b},\mathsf{g}(\mathsf{a}))$ we obtain $\langle s,t,u\rangle = \mathsf{fgf}(\mathsf{ggb}(\mathsf{aa}\bot),\mathsf{f}\bot\mathsf{g}(\mathsf{b}\bot\mathsf{a},\mathsf{b}\bot\bot))$. An $n$-ary relation on ground terms is regular if its encoding is accepted by a tree automaton operating on terms in $\mathcal{T}(\mathcal{F}^{(n)})$. Such an automaton is called an $\mathsf{RR}_n$ automaton and regular $n$-ary relations are called $\mathsf{RR}_n$ relations. The $i$-th cylindrification of an $\mathsf{RR}_n$ relation $R$ over $\mathcal{T}(\mathcal{F})$ is the $\mathsf{RR}_{n+1}$ relation $\{(t_1,\ldots,t_{i-1},u,t_i,\ldots,t_n) \mid (t_1,\ldots,t_n) \in R \text{ and } u \in \mathcal{T}(\mathcal{F})\}$.

Besides $\mathsf{RR}_n$ automata, the decision procedure makes use of ground tree transducers (GTTs for short). A GTT is a pair $\mathcal{G} = (\mathcal{A},\mathcal{B})$ of tree automata over the same signature $\mathcal{F}$. A pair $(s,t)$ of ground terms in $\mathcal{T}(\mathcal{F})$ is accepted by $\mathcal{G}$ if $s \to_\mathcal{A}^* u \;{}_\mathcal{B}^*\!\leftarrow t$ for some term $u \in \mathcal{T}(\mathcal{F} \cup Q)$. Here $Q$ is the combined set of states of $\mathcal{A}$ and $\mathcal{B}$. The set of all such pairs is denoted by $L(\mathcal{G})$. We denote by

$L_a(\mathcal{G})$ the set of all pairs $(s,t)$ such that $s \to_{\mathcal{A}}^* q \; {}_{\mathcal{B}}^{*}\!\!\leftarrow t$ for some state $q \in Q$. A binary relation $R$ on ground terms is a(n anchored) GTT relation if there exists a GTT $\mathcal{G}$ such that $R = L(\mathcal{G})$ ($R = L_a(\mathcal{G})$). The decision procedure for the first-order theory of rewriting described in [7] and implemented in FORT uses GTTs, the formalized variant described in [14] uses anchored GTTs (aGTTs), which have better closure properties. Both are supported in our certificate language, but FORT-h and FORTify use anchored GTTs since they permit us to model more predicates while reducing the need for ad-hoc constructions that need to be turned into executable (verified) code.

The decision procedure for the first-order theory of rewriting constructs $\mathsf{RR}_n$ automata for the subformulas in a bottom-up fashion. GTTs (aGTTs) come into play for some of the atomic subformulas consisting of predicate symbols and variables. Closure properties take care of the logical structure of formulas. A final emptiness check determines whether the formula is satisfied for the TRS given as input to the decision procedure. Rather than formally stating the properties involved, we illustrate the decision procedure on an example.

*Example 1.* Consider the formula $\varphi = \forall\, s\, \exists\, t\, (s \to^* t \wedge \mathsf{NF}(t))$, which expresses the normalization property of TRSs. To determine whether a TRS $\mathcal{R}$ over a signature $\mathcal{F}$ satisfies $\varphi$, we first construct an $\mathsf{RR}_1$ automaton $\mathcal{A}_1$ that accepts the ground normal forms in $\mathcal{T}(\mathcal{F})$, using an algorithm first described in [5] and recently formalized in [13]. For the subformula $s \to^* t$ we construct a GTT $\mathcal{G}_1$ for the parallel rewrite relation $\twoheadrightarrow\!\!\!\!\!\!\;|\;\;_{\mathcal{R}}$. Since GTT relations are effectively closed under transitive closure (while $\mathsf{RR}_2$ relations are not), we obtain a GTT $\mathcal{G}_2$ for $\to_{\mathcal{R}}^*$. This GTT is transformed into an $\mathsf{RR}_2$ automaton $\mathcal{A}_2$. (In the formalized decision procedure described in [14], an $\mathsf{RR}_2$ automaton for $\to^*$ is constructed from an anchored GTT for the root step relation $\to_{\mathcal{R}}^{\epsilon}$, using suitable closure properties of anchored GTT and $\mathsf{RR}_2$ relations.) We cylindrify the $\mathsf{RR}_1$ automaton $\mathcal{A}_1$ into an $\mathsf{RR}_2$ automaton $\mathcal{A}_3$ that accepts $\mathcal{T}(\mathcal{F}) \times \mathsf{NF}_{\mathcal{R}}$. A product construction involving $\mathcal{A}_2$ and $\mathcal{A}_3$ produces an $\mathsf{RR}_2$ automaton $\mathcal{A}_4$ for the subformula $s \to^* t \wedge \mathsf{NF}(t)$. Projection yields an $\mathsf{RR}_1$ automaton $\mathcal{A}_5$ corresponding to $\exists\, t\, (s \to^* t \wedge \mathsf{NF}(t))$. So $\varphi$ holds if and only if $L(\mathcal{A}_5) = \mathcal{T}(\mathcal{F})$. In FORT the $\forall$ quantifier is transformed into the equivalent $\neg\,\exists\,\neg$. Hence complementation is used to obtain an $\mathsf{RR}_1$ automaton $\mathcal{A}_6$ and the existential quantifier is implemented using projection. This gives an $\mathsf{RR}_0$ automaton $\mathcal{A}_7$ which either accepts the empty relation $\varnothing$ or the singleton set $\{()\}$ consisting of the nullary tuple $()$. The outermost negation gives rise to another complementation step. The final $\mathsf{RR}_0$ automaton $\mathcal{A}_8$ is tested for emptiness: $L(\mathcal{A}_8) = \varnothing$ if and only the TRS $\mathcal{R}$ does not satisfy $\varphi$.

## 3 Formulas

The first step in the certification process is to translate formulas in the first-order theory of rewriting into a format suitable for further processing. We adopt de Bruijn indices [4] to avoid alpha renaming.

*Example 2.* Consider the formula

```
forall s, t, u ([0] s ->* t & [1] s ->* u  =>
       exists v ([1] t ->* v & [0] u ->* v))
```

in FORT syntax. It expresses the *commutation* of two TRSs, indicated by the indices 0 and 1. Using de Bruijn indices for the term variables $s$, $t$, $u$, $v$ produces

$$\forall\forall\forall\,(2 \to_0^* 1 \wedge 2 \to_1^* 0) \implies \exists\,(2 \to_1^* 0 \wedge 1 \to_0^* 0)$$

We refer to Example 4 for further explanation.

The formal syntax of formulas in certificates is given below. Angle brackets $\langle\ \rangle$ are used for non-terminal symbols. Here $\langle rr_2 \rangle$ denotes the supported binary regular relations, which are formally defined after Example 3. Likewise, $\langle rr_1 \rangle$ stands for regular sets (which are identified with unary regular relations).

$$\begin{aligned}\langle \mathit{formula} \rangle \ ::=\ & \texttt{(rr1}\,\langle rr_1 \rangle\,\langle \mathit{term} \rangle\texttt{)} \mid \texttt{(rr2}\,\langle rr_2 \rangle\,\langle \mathit{term} \rangle\,\langle \mathit{term} \rangle\texttt{)}\\ & \mid \texttt{(and}\,\langle \mathit{formula} \rangle *\texttt{)} \mid \texttt{(or}\,\langle \mathit{formula} \rangle *\texttt{)} \mid \texttt{(not}\,\langle \mathit{formula} \rangle\texttt{)}\\ & \mid \texttt{(forall}\,\langle \mathit{formula} \rangle\texttt{)} \mid \texttt{(exists}\,\langle \mathit{formula} \rangle\texttt{)} \mid \texttt{(true)} \mid \texttt{(false)}\\ & \mid \texttt{(restrict}\,\langle \mathit{formula} \rangle\,\texttt{(}\,\langle \mathit{trs} \rangle +\texttt{))}\end{aligned}$$

$$\langle \mathit{term} \rangle ::= \langle \mathit{nat} \rangle \qquad \langle \mathit{trs} \rangle ::= \langle \mathit{nat} \rangle \mid \langle \mathit{nat} \rangle\texttt{-} \qquad \langle \mathit{nat} \rangle ::= \texttt{0} \mid \texttt{1} \mid \texttt{2} \mid \cdots$$

De Bruijn indices are used for $\langle \mathit{term} \rangle$ variables and $\langle \mathit{nat} \rangle$`-` denotes a TRS with index $\langle \mathit{nat} \rangle$ in which the left- and right-hand sides of the rules have been swapped. The class of linear variable-separated TRSs is closed under this operation. We use it to represent the conversion relation $\leftrightarrow^*$ of a TRS $\mathcal{R}$ as the reachability relation $\to^*$ induced by the TRS $\mathcal{R} \cup \mathcal{R}^-$.

*Example 3.* The commutation property in Example 2 is rendered as follows:

```
(forall (forall (forall (or (not (and (rr2 (step* (0)) 2 1)
  (rr2 (step* (1)) 2 0))) (exists (and (rr2 (step* (1)) 2 0)
    (rr2 (step* (0)) 1 0)))))))
```

Here `(step* (0))` denotes the $\mathsf{RR}_2$ relation $\to^*$ induced by the first TRS (which is indexed by 0) and `(rr2 (step* (1)) 2 0)` represents the subformula `[1] t ->* v` of the FORT formula in Example 2.

We continue with the certificate syntax of $\mathsf{RR}_1$ and $\mathsf{RR}_2$ relations:

$$\begin{aligned}\langle rr_1 \rangle\ ::=\ & \texttt{(terms)} \mid \texttt{(nf (}\,\langle \mathit{trs} \rangle +\texttt{))} \mid \texttt{(inf}\,\langle rr_2 \rangle\texttt{)} \mid \texttt{(proj}\,\texttt{(1}\,|\,\texttt{2)}\,\langle rr_2 \rangle\texttt{)}\\ & \mid \texttt{(union}\,\langle rr_1 \rangle\,\langle rr_1 \rangle\texttt{)} \mid \texttt{(inter}\,\langle rr_1 \rangle\,\langle rr_1 \rangle\texttt{)} \mid \texttt{(diff}\,\langle rr_1 \rangle\,\langle rr_1 \rangle\texttt{)}\end{aligned}$$

$$\begin{aligned}\langle rr_2 \rangle\ ::=\ & \texttt{(gtt}\,\langle \mathit{gtt} \rangle\,\langle \mathit{pos} \rangle\,\langle \mathit{num} \rangle\texttt{)} \mid \texttt{(product}\,\langle rr_1 \rangle\,\langle rr_1 \rangle\texttt{)} \mid \texttt{(id}\,\langle rr_1 \rangle\texttt{)}\\ & \mid \texttt{(union}\,\langle rr_2 \rangle\,\langle rr_2 \rangle\texttt{)} \mid \texttt{(inter}\,\langle rr_2 \rangle\,\langle rr_2 \rangle\texttt{)} \mid \texttt{(diff}\,\langle rr_2 \rangle\,\langle rr_2 \rangle\texttt{)}\\ & \mid \texttt{(comp}\,\langle rr_2 \rangle\,\langle rr_2 \rangle\texttt{)} \mid \texttt{(inverse}\,\langle rr_2 \rangle\texttt{)}\end{aligned}$$

$$\langle pos \rangle \ ::= \ \texttt{>=} \mid \texttt{e} \mid \texttt{>} \qquad \langle num \rangle \ ::= \ \texttt{>=} \mid \texttt{1} \mid \texttt{>}$$

$$\begin{aligned} \langle gtt \rangle \ ::= \ & \texttt{(root-step (} \langle trs \rangle + \texttt{))} \mid \texttt{(inverse} \langle gtt \rangle \texttt{)} \mid \texttt{(union} \langle gtt \rangle \langle gtt \rangle \texttt{)} \\ & \mid \texttt{(acomp} \langle gtt \rangle \langle gtt \rangle \texttt{)} \mid \texttt{(gcomp} \langle gtt \rangle \langle gtt \rangle \texttt{)} \mid \texttt{(inter} \langle gtt \rangle \langle gtt \rangle \texttt{)} \\ & \mid \texttt{(acomplement} \langle gtt \rangle \texttt{)} \mid \texttt{(atc} \langle gtt \rangle \texttt{)} \mid \texttt{(gtc} \langle gtt \rangle \texttt{)} \end{aligned}$$

Here `(terms)` refers to $\mathcal{T}(\mathcal{F})$, $\texttt{(nf (}\langle trs \rangle + \texttt{))}$ to the normal forms (NF) induced by the union of the underlying TRSs, and $\texttt{(inf}\langle rr_2 \rangle\texttt{)}$ to the infinity predicate ($\mathsf{INF}_R$) which is satisfied by all terms having infinitely many successors with respect to the relation $R$. Furthermore, $\texttt{(proj (1|2)}\langle rr_2 \rangle\texttt{)}$ denotes projection ($\pi$) to the first (second) argument, $\texttt{(gtt}\langle gtt \rangle \langle pos \rangle \langle num \rangle\texttt{)}$ the transformation of a GTT relation into an $\mathsf{RR}_2$ relation with corresponding context closure (cf. [14, Section 3]), $\texttt{(id}\langle rr_1 \rangle\texttt{)}$ the identity relation on the underlying set, and $\texttt{(gtc}\langle gtt \rangle\texttt{)}$ ($\texttt{(atc}\langle gtt \rangle\texttt{)}$) the (anchored) transitive closure of the underlying (anchored) GTT relation.

The constructs defined above closely correspond to the formalized closure operations for the predicates in the first-order theory of rewriting, reported in [14] and summarized below:

$$\begin{aligned} A \ &::= \ \rightarrow_\epsilon \mid A^- \mid A \cup A \mid A^+ \mid A^{\widehat{+}} \mid A \circ A \mid A \mathbin{\widehat{\circ}} A \mid A^c \mid A \cap A \\ R \ &::= \ A \mid R_p^n \mid R \cup R \mid R \cap R \mid R^- \mid T \times T \mid =_T \\ T \ &::= \ \mathcal{T}(\mathcal{F}) \mid \mathsf{NF} \mid \mathsf{INF}_R \mid T \cup T \mid T \cap T \mid T^c \mid \pi_1(R) \mid \pi_2(R) \\ n \ &::= \ \geqslant \mid 1 \mid > \qquad p \ ::= \ \geqslant \mid \epsilon \mid > \end{aligned}$$

Here $A$ are anchored GTT relations ($\langle gtt \rangle$), $R$ are $\mathsf{RR}_2$ relations ($\langle rr_2 \rangle$), and $T$ are regular sets of ground terms ($\langle rr_1 \rangle$).

For convenience of tool authors, we add a few other constructs to $\langle rr_2 \rangle$. The certifier expands these to a sequence of basic constructs given above.

$$\begin{aligned} \langle rr_2 \rangle \ ::= \ & \cdots \mid \texttt{(step (}\langle trs \rangle + \texttt{))} \mid \texttt{(step= (}\langle trs \rangle + \texttt{))} \\ & \mid \texttt{(step+ (}\langle trs \rangle + \texttt{))} \mid \texttt{(step* (}\langle trs \rangle + \texttt{))} \mid \texttt{(equality)} \\ & \mid \texttt{(parallel-step (}\langle trs \rangle + \texttt{))} \mid \texttt{(root-step+ (}\langle trs \rangle + \texttt{))} \\ & \mid \texttt{(non-root-step (}\langle trs \rangle + \texttt{))} \mid \texttt{(join (}\langle trs \rangle + \texttt{))} \end{aligned}$$

The complete list can be obtained from the accompanying website.

## 4 Certificates

A certificate for a first-order formula $\varphi$ explains how the corresponding $\mathsf{RR}_n$ automaton is constructed. We adopt a line-oriented natural deduction style. The automata are implicit. This is a deliberate design decision to keep certificates small. More importantly, it avoids having to check equivalence of finite tree automata, which is EXPTIME-complete [6, Section 1.7].

$$\langle certificate \rangle \ ::= \ \texttt{(} \langle item \rangle \langle inference \rangle \langle formula \rangle \langle info \rangle * \texttt{)} \langle certificate \rangle$$

$$
\begin{aligned}
&\qquad\qquad\quad \mid \texttt{(empty}\,\langle \mathit{item}\rangle\texttt{)} \mid \texttt{(nonempty}\,\langle \mathit{item}\rangle\texttt{)}\\
\langle \mathit{item}\rangle &::= \langle \mathit{nat}\rangle \qquad \langle \mathit{info}\rangle \ ::= \ \texttt{(size}\,\langle \mathit{nat}\rangle\,\langle \mathit{nat}\rangle\,\langle \mathit{nat}\rangle\texttt{)} \mid \cdots\\
\langle \mathit{inference}\rangle &::= \texttt{(rr1}\,\langle \mathit{rr}_1\rangle\,\langle \mathit{term}\rangle\texttt{)} \mid \texttt{(rr2}\,\langle \mathit{rr}_2\rangle\,\langle \mathit{term}\rangle\,\langle \mathit{term}\rangle\texttt{)}\\
&\quad \mid \texttt{(and}\,\langle \mathit{item}\rangle * \texttt{)} \mid \texttt{(or}\,\langle \mathit{item}\rangle * \texttt{)} \mid \texttt{(not}\,\langle \mathit{item}\rangle\texttt{)}\\
&\quad \mid \texttt{(exists}\,\langle \mathit{item}\rangle\texttt{)} \mid \texttt{(nnf}\,\langle \mathit{item}\rangle\texttt{)} \mid \cdots
\end{aligned}
$$

Currently the $\langle \mathit{info}\rangle$ field only serves as an interface between the tool (which provides the certificate) and the certifier to compare the sizes of the constructed automata. In the future we plan to extend this field with concrete automata. This allows to test language equivalence of a tree automaton computed by a tool that supports our certificate language and the one reconstructed by FORTify, thereby providing tool authors with a mechanism to trace buggy constructions in case a certificate is rejected.

We revisit Example 1 to illustrate the construction of certificates.

*Example 4.* The formula $\varphi = \forall\, s\, \exists\, t\, (s \to^* t \wedge \mathsf{NF}(t))$ expressing normalization is rendered as $\varphi' = \forall\exists(1 \to_0^* 0 \wedge 0 \in \mathsf{NF}[0])$ in de Bruijn notation. Here 1 refers to the variable $s$, the second and third occurrences of 0 refer to $t$, and the last occurrence of 0 refer to the first (and only) TRS, which has index 0. We construct the certificate bottom-up, to mimic the decision procedure. The first line is for $\mathsf{NF}[0]$:

```
(0 (rr1 (nf (0)) 0) (rr1 (nf (0)) 0))
```

The components can be read as follows:

- $\langle \mathit{item}\rangle$ = `0` denotes the first step in our proof,
- $\langle \mathit{inference}\rangle$ = `rr1 (nf (0)) 0` construct the automaton that accepts the normal forms and keeps track of the variable 0,
- $\langle \mathit{formula}\rangle$ = `rr1 (nf (0)) 0` denotes the subformula $0 \in \mathsf{NF}[0]$; it is satisfiable if and only if the automaton constructed using the description in $\langle \mathit{inference}\rangle$ is not empty.

The apparent redundancy will disappear when we continue. We proceed by expressing the relation $\to_0^*$ and subsequently make sure that the second component of $\to_0^*$ is in normal form:

```
(1 (rr2 (step* (0)) 1 0) (rr2 (step* (0)) 1 0))
(2 (and (1 0)) (and ((rr2 (step* (0)) 1 0) (rr1 (nf (0)) 0))))
```

Line 1 is similar to line 0. The inference step `and 1 0` in line 2 constructs an $\mathsf{RR}_2$ automaton that accepts the intersection of the relations modeled in lines 1 and 0. This automaton corresponds to $\mathcal{A}_4$ in Example 1. The cylindrification step from $\mathcal{A}_1$ to $\mathcal{A}_3$ in Example 1 is left implicit. We continue with the projection of variable 0 and afterwards complement the resulting automaton. This is done by an `exists` followed by a `not` inference step:

```
(3 (exists 2) (exists (and ((rr2 (step* (0)) 1 0)
   (rr1 (nf (0)) 0)))))
(4 (not 3) (not (exists (and ((rr2 (step* (0)) 1 0)
   (rr1 (nf (0)) 0))))))
```

The inference steps until this point describe the construction of $\mathcal{A}_6$ in Example 1. We complete the certificate by introducing the remaining operators:

```
(5 (exists 4) (exists (not (exists (and ((rr2 (step* (0)) 1 0)
   (rr1 (nf (0)) 0)))))))
(6 (not 5) (not (exists (not (exists (and ((rr2 (step* (0)) 1 0)
   (rr1 (nf (0)) 0))))))))
(7 (nnf 6) (forall (exists (and ((rr2 (step* (0)) 1 0)
   (rr1 (nf (0)) 0))))))
(nonempty 7)
```

The `nnf` inference step does not modify the tree automaton computed in step 6 (which corresponds to $\mathcal{A}_8$ in Example 1) but checks the equivalence of the formula in line 6 with the one of line 7, which corresponds to the input formula $\varphi'$. The equivalence check incorporates $\forall$ elimination, negation normal form, and associativity, commutativity and idempotency of $\wedge$ and $\vee$. In the future we might add support for additional equivalences in first-order logic. The final step `(nonempty 7)` checks that $L(\mathcal{A}_8) \neq \varnothing$. So this certificate claims that the input TRS is normalizing. For TRSs that do not satisfy $\varphi$, the final line in the certificate would be `(empty 7)`.

In the previous example we intentionally skipped over some details to convey the underlying intuition. First of all, the $\langle rr_2 \rangle$ construct `(step* (0))` is derived and internally unfolded via (anchored) GTTs into

```
(gtt (gtc (root-step 0)) >= >)
```

Starting from an anchored GTT that accepts the root step relation induced by the first (and only) TRS in the list, an application of the GTT transitive closure operation followed by a multi-hole context closure operation with at least one hole that may appear in any position, an $\mathsf{RR}_2$ automaton that accepts the relation $\rightarrow_0^*$ is constructed. We also mentioned that cylindrification is implicit. The same holds for the projection operation that is used in the `exists` inference steps. A projection takes place in the first component if the variable 0 is present in the list of variables, otherwise the inference step preserves the automaton. This approach is sound as variables indicate the relevant components of the $\mathsf{RR}_n$ automaton. Thanks to the de Bruijn representation, the innermost quantifier refers to variable 0, the first component in the given $\mathsf{RR_n}$ automaton. However we must keep track of all variables occurring in the surrounding formula and update that list accordingly.

## 5 FORTify

The example in the preceding section makes clear that a certificate can be viewed as a recipe for the certifier to perform certain operations on automata and formulas to confirm the final (non-)emptiness claim. In particular, checking a certificate is expensive because the decision procedure for the first-order theory is replayed using code-generated operations from a verified version of the decision procedure. In this section we describe the steps we performed to turn the Isabelle formalization of the decision procedure described in [14] into our certifier FORTify.

We use the FOL-Fitting library [3], which is part of the Archive of Formal Proofs,[4] to connect the first-order theory of rewriting and first-order logic. The translation is more or less straightforward. We interpret $\mathsf{RR}_1$ constructions as predicates and $\mathsf{RR}_2$ construction as relations in first-order logic and prove both interpretations to be semantically equivalent:

**lemma** *eval_formula* $\mathcal{F}$ *Rs* $\alpha$ *f* =
*eval* $\alpha$ *undefined* (*for_eval_rel* $\mathcal{F}$ *Rs*) (*form_of_formula* *f*)

With this equivalence we are able to define the semantics of formulas:

**definition** *formula_satisfiable* **where**
*formula_satisfiable* $\mathcal{F}$ *Rs f* $\longleftrightarrow$ ($\exists\,\alpha$. *range* $\alpha \subseteq \mathcal{T}_G\ \mathcal{F}$ $\wedge$
*eval_formula* $\mathcal{F}$ *Rs* $\alpha$ *f*)

**definition** *formula_unsatisfiable* **where**
*formula_unsatisfiable* $\mathcal{F}$ *Rs fm* $\longleftrightarrow$ (*formula_satisfiable* $\mathcal{F}$ *Rs fm* = *False*)

**definition** *correct_certificate* **where**
*correct_certificate* $\mathcal{F}$ *Rs claim infs n* $\equiv$
(*claim* = *Empty* $\longleftrightarrow$ (*formula_unsatisfiable* (*fset* $\mathcal{F}$) (*map fset Rs*)
(*fst* (*snd* (*snd* (*infs* ! *n*))))) $\wedge$
*claim* = *Nonempty* $\longleftrightarrow$ *formula_satisfiable* (*fset* $\mathcal{F}$) (*map fset Rs*)
(*fst* (*snd* (*snd* (*infs* ! *n*)))))

Last but not least we define the important function `check_certificate` which takes as input a signature, a list of TRSs, a boolean, a formula, and a certificate. This function first verifies that the given formula and the claim corresponds to the ones referenced in the certificate and afterwards checks the integrity of the certificate. The following lemmata, which are formally proved in Isabelle, state the correctness of the `check_certificate` function:

**lemma** *check_certificate* $\mathcal{F}$ *Rs A fm* (*Certificate infs claim n*) = *Some B*
$\Longrightarrow$ *fm* = *fst* (*snd* (*snd* (*infs* ! *n*))) $\wedge$ *A* = (*claim* = *Nonempty*)

**lemma** *check_certificate* $\mathcal{F}$ *Rs A fm* (*Certificate infs claim n*) = *Some B*
$\Longrightarrow$ (*B* = *True* $\longrightarrow$ *correct_certificate* $\mathcal{F}$ *Rs claim infs n*) $\wedge$
(*B* = *False* $\longrightarrow$ *correct_certificate* $\mathcal{F}$ *Rs* (*case claim of*
*Empty* $\Rightarrow$ *Nonempty* | *Nonempty* $\Rightarrow$ *Empty*) *infs n*)

[4] https://www.isa-afp.org

The first lemma ensures that our check function verifies that the provided parameters *fm* (formula) and *A* (answer satisfiable/unsatisfiable) match the formula and the claim stated in the certificate. The second lemma is the key result. It states that the check function returns `Some True` if and only if the certificate is correct. The only-if case is hidden in the last two lines. More precisely, if the claim of the certificate is wrong then negating the claim (the first-order theory of rewriting is complete) leads to a correct certificate. Therefore, if our check function returns `Some None` then the certificate is correct after negating the claim.

Our check function returns `None` if the global assumptions (the input TRS is not linear variable-separated, the signature is not empty, etc.) are not fulfilled. We plan to extend the `check_certificate` function in the near future such that it reports these kind of errors.

A central part of the formalization is to obtain a trustworthy decision procedure to verify certificates. Hence we use the code generation facility of Isabelle/HOL to produce an executable version of our `check_certificate` function. Isabelle's code generation facility is able to derive executable code for our constructions with the exception of inductively defined sets. In [8, Section 7] an abstract *Horn inference system* for finite sets is introduced to overcome this limitation. We use this framework to obtain executable code for the following constructions defined as inductive sets:

- reachable and productive states of a tree automaton,
- states of tree automata obtained by the subset construction,
- epsilon transitions for the composition and transitive closure constructions of (anchored) GTTs,
- an inductive set needed for the tree automaton for the infinity predicate.

At this point we can use Isabelle's code generation to obtain an executable check function. However, more effort is needed to obtain an efficient check function. Checking the certificate in Example 6 below did not terminate after more than 24 hours computation time. We used the profiling capabilities of the Glasgow Haskell Compiler (GHC) to analyze the generated code. This revealed that most of the time was spent on checking membership. Since the computed tree automata can grow very large, the use of lists as underlying data structure for sets in the generated code is a bottleneck.

To overcome this problem we decided to use the container framework of Lochbihler [12]. In our case, the setup involved a non-trivial overhead as the container framework requires multiple class instances for data types used inside sets. Some of these instances could be derived automatically by the deriving framework of Sternagel and Thiemann [20]. Afterwards Isabelle's code generation was able to generate a `check_certificate` function that uses red-black trees as underlying data structure for sets.

Sadly, the function was still infeasible for the certificate in Example 6. This time the power set construction, which is exponential in worst case, turned out to be the culprit. In this construction we compute the transitive closure of the present epsilon transitions multiple times. Adding an explicit construction to

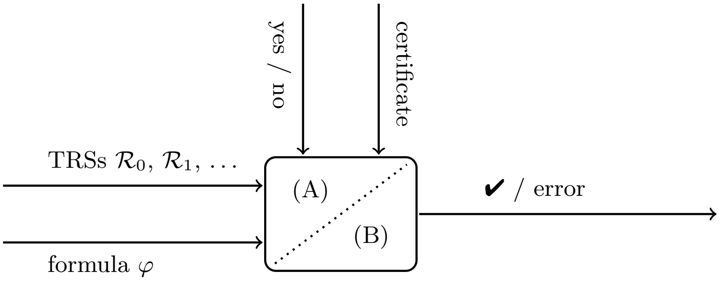

**Fig. 1.** Certificate validation with FORTify.

remove epsilon transitions from tree automata solved this issue. To make a long story short, after further modifications we were able to verify the certificate for Example 6 in a little less than 3 minutes, which we consider fast enough for a first prototype. The resulting code-generated certifier is called FORTify.

The overall design of FORTify is shown in Figure 1. It can be viewed as two separate modules A and B. Module B is the verified Haskell code base that is generated by Isabelle's code generation facility, containing the `check_certificate` function and the data type declarations for formulas and certificates. To use this functionality, we wrote a parser which translates strings representing formulas (signatures, TRSs, certificates) to semantically equivalent formulas (signatures, TRSs, certificates) represented in the data types obtained from the generated code. This was done in Haskell and refers to module A in Figure 1. Module A accepts formulas in FORT syntax. Hence it also applies the conversion to the de Bruijn representation. After the translation in module A, the `check_certificate` function in module B is executed and its output is reported.

Importantly, the code in module A is not verified in Isabelle. Correctness of FORTify must therefore assume correctness of module A as well as the correctness of the Glasgow Haskell Compiler, which we use to generate a standalone executable from the generated code.

## 6 FORT-h

FORT-h is a new decision tool for the first order theory of rewriting. It is a reimplementation of the decision mode of the previous FORT tool [18] based on a modified decision procedure. The decision procedure, like the formalization, is based on anchored GTTs. The new tool is implemented in Haskell whereas FORT is written in Java.

FORT-h supports all features of FORT while extending the domain of supported TRSs from left-linear right-ground TRSs to *linear variable-separated* ones. While FORT could technically take such TRSs as input, it is unsound when checking non-ground properties on them.

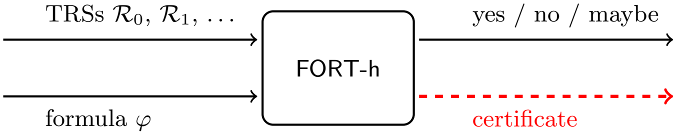

**Fig. 2.** Interface of FORT-h.

*Example 5.* To check confluence of the linear variable-separated TRS

$$\mathsf{g}(\mathsf{g}(x)) \to \mathsf{g}(y) \qquad\qquad \mathsf{a} \to \mathsf{g}(\mathsf{a})$$

FORT-h can be called with

```
> ./fort-h "CR" input.trs
NO
```

where `input.trs` is a text file containing the rewrite system. The tool correctly states that NO the system is not confluent. However, FORT incorrectly identifies this as confluent due to the lack of support for variables appearing in right-hand sides of rules.

FORT-h took part in the 2020 edition of the Confluence Competition, competing in five categories: COM, GCR, NFP, UNC and UNR. Even though it does not support many problems tested in the competition, due to the restriction to linear variable-separated TRSs, it was able to win the category for most YES results in UNR. The tool expects as input a formula $\varphi$ and one or more TRSs, as seen in Figure 2. It then outputs the answer YES or NO depending on whether $\varphi$ is satisfied or not by the given TRSs. FORT-h may be passed some additional options:

**`-c FILE`:** causes FORT-h to write a certificate to the given `FILE`,
**`-i`:** enables the additional ⟨*info*⟩ in the inference steps in the certificate,
**`-v`:** enables verbose output (e.g. showing the internal formula representation).
**`-w`:** enables witness generation.

As an example of the latter, consider Example 5 and the call

```
> fort-h -w "CR" input.trs
NO
formula body / witness:
    (0 (<- o ->*) 1 & ~ 0 (->* o *<-) 1)
    0 = g(_00())
    1 = g(_01())
```

So in addition to the answer NO, it also outputs a counter example for the given formula consisting of the two terms `g(_00())` and `g(_01())`. Here `_00` and `_01`

are additional constants required to reduce confluence to ground-confluence, and represent variables. The terms should therefore be read as $\mathsf{g}(x)$ and $\mathsf{g}(y)$.

Internally FORT-h represents formulas using de Bruijn indices as described in Section 4. Additionally, universal quantifiers and implications are eliminated, and negations are pushed as far as possible to the atomic subformulas. The tool then traverses the formula in a bottom-up fashion, constructing the corresponding anchored GTTs and $\mathsf{RR}_n$ automata. During this traversal we also keep track of the steps taken, to construct the certificate if necessary. To improve performance the automata are cached and reused for equal subformulas. The tree automaton representing the whole formula is then checked for emptiness. If the accepted language is empty, FORT-h reports NO, otherwise it outputs YES.

## 7 Experiments

The experiments described in this section were run on a computer with a Intel(R) Core(TM) i7-5930K CPU with 6 cores at 3.50GHz.

In the 2019 edition of the Confluence Competition [15] three tools contested the commutation (COM) category:[5] ACP [1], CoLL [19], and FORT. On input problem COPS #1118 the tools gave conflicting answers.

*Example 6.* COPS #1118 is about the commutation of the TRSs COPS #669

$$\mathsf{a} \to \mathsf{c} \qquad \mathsf{f}(\mathsf{a}) \to \mathsf{b} \qquad \mathsf{b} \to \mathsf{b} \qquad \mathsf{b} \to \mathsf{h}(\mathsf{b}, \mathsf{h}(\mathsf{c}, \mathsf{a}))$$

and COPS #695

$$\mathsf{h}(\mathsf{a}, \mathsf{a}) \to \mathsf{c} \qquad \mathsf{b} \to \mathsf{h}(\mathsf{b}, \mathsf{a}) \qquad \mathsf{b} \to \mathsf{a} \qquad \mathsf{f}(\mathsf{c}) \to \mathsf{c} \qquad \mathsf{c} \to \mathsf{a}$$

To determine the correct answer we use FORT-h to produce a certificate for ground-confluence by calling

```
> fort-h -c cert -i "GCom([0],[1])" 1118.trs
YES
```

This produces the following certificate:

```
(0 (rr2 (comp (inverse (step* (1))) (step* (0))) 0 1)
   (rr2 (comp (inverse (step* (1))) (step* (0))) 0 1)
   (size 13 53 0))
(1 (rr2 (comp (step* (0)) (inverse (step* (1)))) 0 1)
   (rr2 (comp (step* (0)) (inverse (step* (1)))) 0 1)
   (size 11 47 0))
(2 (not 1) (not (rr2 (comp (step* (0)) (inverse (step* (1)))) 0 1)))
(3 (and (0 2))
   (and ((rr2 (comp (inverse (step* (1))) (step* (0))) 0 1)
      (not (rr2 (comp (step* (0)) (inverse (step* (1)))) 0 1)))))
(4 (exists 3)
```

[5] https://cops.uibk.ac.at/results/?y=2019&c=COM

**Table 1.** FORT(-h) run on GCR formulas with a 60 s timeout (FORTify with 600 s).

| | | YES | ∅-time | ✔ | NO | ∅-time | ✔ | ∞ | total (✔) time | |
|---|---|---|---|---|---|---|---|---|---|---|
| (1) | FORT-h | 36 | 0.26 s | 10 | 84 | 0.56 s | 16 | 2 | 176.23 s | (17.6 h) |
| | FORT | 37 | 0.31 s | — | 82 | 0.52 s | — | 3 | 234.08 s | |
| (2) | FORT-h | 37 | 1.48 s | 10 | 84 | 0.09 s | 16 | 1 | 122.55 s | (17.8 h) |
| | FORT | 37 | 0.32 s | — | 82 | 0.50 s | — | 3 | 233.20 s | |
| (3) | FORT-h | 36 | 0.45 s | 6 | 83 | 0.08 s | 9 | 3 | 202.64 s | (18.2 h) |
| | FORT | 37 | 0.32 s | — | 82 | 0.55 s | — | 3 | 236.69 s | |

```
   (exists (and ((rr2 (comp (inverse (step* (1))) (step* (0))) 0 1)
     (not (rr2 (comp (step* (0)) (inverse (step* (1)))) 0 1))))))
(5 (exists 4)
   (exists (exists (and ((rr2 (comp (inverse (step* (1)))
     (step* (0))) 0 1) (not (rr2 (comp (step* (0))
     (inverse (step* (1)))) 0 1)))))))
(6 (not 5)
   (not (exists (exists (and (
     (rr2 (comp (inverse (step* (1))) (step* (0))) 0 1)
     (not (rr2 (comp (step* (0)) (inverse (step* (1)))) 0 1))))))))
(7 (nnf 6)
   (forall (forall (or (
     (not (rr2 (comp (inverse (step* (1))) (step* (0))) 0 1))
     (rr2 (comp (step* (0)) (inverse (step* (1)))) 0 1))))))
(nonempty 7)
```

When passing this certificate to FORTify, after 2 minutes and 57 seconds the output `Certified` is produced, so we can be assured that the TRSs do commute. Note that the inference steps `0` and `1` contain the optional size information. Here `(size k m n)` means the underlying $\mathsf{RR}_n$ automaton constructed by FORT-h contains `k` final states, `m` transitions, and `n` epsilon transitions.

We also ran some experiments comparing FORT-h to FORT. The problems for these experiments are taken from the Confluence Problems database (COPS), and consists of 122 left-linear right-ground TRSs. Note that FORT-h implements no parallelism, while FORT does. For the first two experiments we chose a timeout of 60 seconds for the decision tools and 600 seconds for FORTify. The formulas were taken from the experiments reported in [17]. The first three

$$\forall\, s\, \forall\, t\, \forall\, u\, (s \to^* t \wedge s \to^* u \implies t \downarrow u) \tag{1}$$

$$\forall\, s\, \forall\, t\, \forall\, u\, (s \to^* t \wedge s \to u \implies t \downarrow u) \tag{2}$$

$$\forall\, t\, \forall\, u\, (t \leftrightarrow^* u \implies t \downarrow u) \tag{3}$$

denote different but equivalent formulations of ground-confluence (GCR).

The results are shown in Table 1, where the YES (NO) column shows the number of systems determined to be (non-)ground-confluent together with average time (∅-time) the tool took. The ∞ column is the number of timeouts.

To compare overall performance the *total time* column contains the sum of all runtimes, including timeouts but excluding the time taken by FORTify. The ✔ columns show the numbers of certifiable results as well as the overall time taken by FORTify (✔-time). These results show that, even though they have the same meaning, the choice of formula has an impact on performance. Interestingly FORT-h is generally faster and can solve more problems than FORT even though it can not take advantage of any parallelism. This performance advantage is more prominent in systems which are non-confluent. For problems with the answer YES, FORT can still prove more. The table also shows that FORTify can only certify a small portion the results. This is due to the performance of the certifier, since all other problems time out. It is also apparent that formulas containing conversion ($\leftrightarrow^*$) are especially slow. No wrong results by the decision tools where identified.

The second set of formulas represents the normal form property, restricted to ground terms (GNFP):

$$\forall\, t\, \forall\, u\, (t \leftrightarrow^* u \wedge \mathsf{NF}(u) \implies t \to^* u) \tag{4}$$

$$\forall\, s\, \forall\, t\, \forall\, u\, (s \to t \wedge s \to^! u \implies t \to^* u) \tag{5}$$

$$\forall\, t\, (\mathsf{WN}(t) \implies \mathsf{CR}(t)) \tag{6}$$

The results for these are shown in Table 2. The same pattern is observed, where even though both can (dis)prove satisfaction for the same formulas, FORT-h is faster overall.

For the last experiment we test performance on properties over two TRSs. This is done by checking ground-commutation (GCOM) for all pairs of systems form the dataset, resulting in 7503 problems. A timeout of 60 seconds was used. The results, presented in Table 3, show that FORT-h is ahead here as well, (dis)proving more problems and doing so in significantly less time.

Full details of the experiments are available from the website[6] accompanying this paper. Precompiled binaries of FORT-h and FORTify are available from the same site. We also present a few additional experiments with FORTify.

[6] https://fortissimo.uibk.ac.at/tacas2021

**Table 2.** FORT(-h) run on GNFP formulas with a 60 s timeout (FORTify with 600 s).

| | | YES | ∅-time | ✔ | NO | ∅-time | ✔ | ∞ | total | (✔) time |
|---|---|---|---|---|---|---|---|---|---|---|
| (4) | FORT-h | 59 | 0.70 s | 31 | 63 | 0.07 s | 20 | 0 | 45.62 s | (14.6 h) |
| | FORT | 59 | 0.23 s | — | 63 | 0.39 s | — | 0 | 38.16 s | |
| (5) | FORT-h | 59 | 0.03 s | 46 | 63 | 0.01 s | 50 | 0 | 2.55 s | (6.3 h) |
| | FORT | 59 | 0.22 s | — | 63 | 0.30 s | — | 0 | 31.83 s | |
| (6) | FORT-h | 59 | 0.05 s | 42 | 62 | 0.12 s | 45 | 1 | 70.51 s | (8.6 h) |
| | FORT | 59 | 0.31 s | — | 62 | 0.64 s | — | 1 | 117.86 s | |

**Table 3.** FORT(-h) run on GCOM with a 60 s timeout (FORTify with 600 s).

| | YES | ∅-time | ✔ | NO | ∅-time | ✔ | ∞ | total | (✔) time |
|---|---|---|---|---|---|---|---|---|---|
| FORT-h | 1381 | 0.16 s | 878 | 6120 | 0.03 s | 3666 | 2 | 517.32 s | (681.5 h) |
| FORT | 1354 | 1.46 s | — | 6100 | 0.94 s | — | 49 | 10670.89 s | |

## 8 Conclusion

In this paper we presented FORTify, a certifier for the first-order theory of rewriting for linear variable-separated TRSs, together with an expressive certificate language for formulas and proofs. Moreover, a new implementation of the decision procedure for the theory of rewriting, FORT-h, is capable of producing certificates in this language.

We mention three topics which require further research. First of all, many certificates produced by FORT-h cannot be validated by the current version of FORTify within reasonable time. We will further improve the algorithms and data structures used in the `check-certificate` function. A natural candidate for optimization is the transitive closure algorithm generated by Isabelle, which always takes cubic time. Currently, sharing only takes place in the inference rules. Expanding this to the individual constructions will be the next step. Also trimming of anchored GTTs could improve the run time. In the current state of the formalization only trimming of GTTs is proved to be sound. Profiling will be used to determine other candidates that are likely to have a large impact on the validation time.

A second topic for future research is the certification of properties on open (i.e., non-ground) terms. In [8,16,18] conditions are presented to reduce properties related to confluence to the corresponding properties on ground terms, by adding additional constants to the signature. These results need to be formalized in Isabelle and the certificate language needs to be extended, before FORTify can be used to certify the corresponding categories in the Confluence Competition. We plan to define signature extensions directly in formulas, to offer the most flexibility. A related issue is the support for many-sorted signatures in the Isabelle formalization. FORT-h already supports many-sorted TRSs, which is the format in the GCR category of CoCo.

A third topic is improving the efficiency of FORT-h. We anticipate that supporting parallelism will further speed up FORT-h, especially for large formulas. Preprocessing techniques that go beyond the mere transformation to negation normal form will be helpful to obtain equivalent formulas that reduce the size of the ensuing tree automata in the decision procedure. In [10] similar ideas are applied to WS$k$S, in connection with MONA [11].

*Acknowledgments.* We thank René Thiemann for giving valuable advice on how to improve the efficiency of the generated code. The comments by the anonymous reviewers improved the presentation.
